# Case Report: Radiologic disappearance of an incarcerated obturator hernia on sequential CT imaging before elective laparoscopic TAPP repair

**DOI:** 10.3389/fsurg.2026.1887015

**Published:** 2026-07-17

**Authors:** Vu Trung Truc, Ho Trong Thuong, Tran Kien Quyet, Tran Trung Dung, Nguyen Duc Long

**Affiliations:** Digestive System Surgery Department, Saint Paul General Hospital, Hanoi, Vietnam

**Keywords:** case report, laparoscopic TAPP repair, obturator hernia, pelvic hernia, sequential computed tomography, spontaneous reduction

## Abstract

Obturator hernia (OH) is a rare but potentially life-threatening pelvic hernia associated with delayed diagnosis, bowel strangulation, and high mortality. We present a rare case of spontaneously reduced right obturator hernia documented by sequential computed tomography (CT) imaging and successfully treated with elective laparoscopic transabdominal preperitoneal (TAPP) mesh repair. A 67-year-old thin woman presented with acute lower abdominal pain associated with medial right thigh pain consistent with a positive Howship–Romberg sign. Initial non-contrast CT performed at a district hospital demonstrated a right obturator hernia with incarcerated bowel protruding through the obturator canal. However, following interhospital transfer, the patient experienced complete spontaneous symptom resolution, and repeat contrast-enhanced CT no longer demonstrated the herniated bowel loop or evidence of bowel obstruction. Despite apparent clinical and radiologic improvement, the previously documented obturator defect was considered at high risk for recurrent incarceration and strangulation. Elective laparoscopic TAPP repair performed three days later confirmed a widened right obturator defect without residual incarcerated bowel, and prosthetic mesh reinforcement was completed successfully without bowel resection or perioperative complications. The postoperative course was uneventful. This case highlights the diagnostic challenge of spontaneously reduced obturator hernia and emphasizes the importance of correlating prior imaging findings with clinical evolution, even when repeat imaging appears normal. Sequential CT imaging uniquely documented the dynamic transition from incarcerated obturator hernia to spontaneous reduction, thereby preventing missed diagnosis through careful correlation of prior and repeat imaging findings despite complete symptom resolution.

## Introduction

Obturator hernia (OH) is an uncommon pelvic floor hernia in which intra-abdominal contents protrude through the obturator canal (1, 2). It accounts for less than 1% of all abdominal wall hernias and predominantly affects elderly, emaciated, multiparous women because of progressive loss of preperitoneal fat and laxity of pelvic tissues with aging (1–4). Despite its rarity, OH is clinically important because it carries the highest mortality rate among abdominal wall hernias, mainly owing to delayed diagnosis and the high risk of bowel strangulation and ischemia (2–4). Clinical presentation is often nonspecific and may mimic musculoskeletal or nonsurgical abdominal conditions, leading to missed or late recognition (5, 6). The classic Howship–Romberg sign, characterized by pain radiating along the medial thigh due to obturator nerve compression, is considered suggestive but is inconsistently present (5). In contemporary practice, computed tomography (CT) has become the diagnostic modality of choice because of its high sensitivity for identifying herniated bowel within the obturator canal and its ability to detect early bowel compromise before irreversible ischemia develops (3, 5).

Emergency laparotomy has traditionally been the standard surgical approach for strangulated OH, particularly in patients presenting with bowel obstruction or bowel necrosis (1, 2). However, advances in minimally invasive surgery have increasingly favored laparoscopic techniques, especially the transabdominal preperitoneal (TAPP) approach, because of improved visualization of the pelvic floor, evaluation of contralateral defects, reduced postoperative pain, and shorter hospital stay (7). Nevertheless, spontaneous reduction of an obturator hernia before definitive surgical intervention remains distinctly uncommon and may create substantial diagnostic uncertainty, especially when repeat imaging no longer demonstrates the herniated bowel loop. Failure to recognize this phenomenon may result in underdiagnosis and subsequent recurrent incarceration. Herein, we report a rare case of spontaneously reduced right obturator hernia diagnosed initially on CT imaging and subsequently managed successfully with elective laparoscopic TAPP mesh repair. This case highlights the importance of correlating prior imaging findings with clinical evolution and supports definitive minimally invasive repair even after spontaneous reduction has occurred.

## Case presentation

A 67-year-old woman was referred to our emergency department with acute lower abdominal pain of approximately 24 h’ duration. The pain had started suddenly, was colicky in nature, and was predominantly located in the hypogastric region. Notably, the patient also reported radiation of pain to the medial aspect of the right thigh, which was exacerbated during episodes of abdominal pain. She had no vomiting, abdominal distension, obstipation, or inability to pass flatus at the time of presentation. Her medical history was unremarkable for previous abdominal surgery or known abdominal wall hernia. She had previously delivered three children vaginally and reported no family history of abdominal wall or obturator hernia. No psychosocial factors relevant to the present illness were identified. On physical examination, she was alert and haemodynamically stable. Her body habitus was relatively thin, with a body mass index of 19.47 kg/m^2^. Abdominal examination revealed mild tenderness in the lower abdomen without guarding, rebound tenderness, visible peristalsis, or palpable abdominal mass. A clinically significant Howship–Romberg sign was present on the right side, characterized by pain radiating along the medial thigh, raising suspicion for obturator nerve irritation. Initial laboratory investigations demonstrated a white blood cell count of 8.5 G/L with neutrophil predominance, mild anemia with a hemoglobin level of 107 g/L, and a platelet count of 184 G/L. Coagulation parameters were within normal limits, with a prothrombin time international normalized ratio of 1.02 and an activated partial thromboplastin time of 27.6 s. Serum creatinine, electrolytes, and cardiac troponin were not suggestive of acute renal, electrolyte, or myocardial injury. These findings did not indicate severe systemic inflammatory response or advanced bowel ischemia at the time of reassessment.

Before referral, the patient had undergone non-contrast abdominal computed tomography at a district hospital because of persistent abdominal pain and concern for early intestinal obstruction. The initial CT scan demonstrated a right-sided obturator hernia, with a herniated bowel segment passing through the right obturator canal and located between the pectineus muscle anteriorly and the obturator externus muscle posteriorly (Figure 1). This anatomical location was considered highly suggestive of obturator hernia. Given the narrow and rigid nature of the obturator canal and the potential risk of strangulation, the patient was initially considered for emergency surgical management.

**Figure 1:**
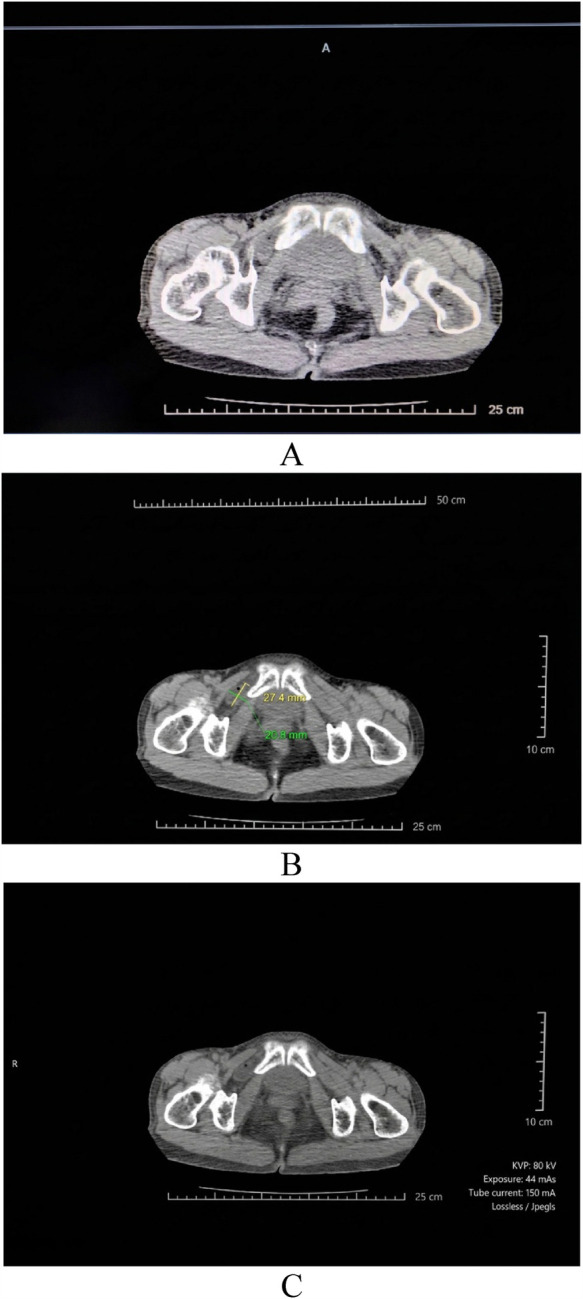
Initial sequential non-contrast CT images documenting incarceration before spontaneous reduction. **(A)** Axial CT image showing bowel protrusion through the right obturator canal between the pectineus muscle anteriorly and the obturator externus muscle posteriorly. **(B)** Axial CT image with radiologic measurements demonstrating the incarcerated bowel segment within the right obturator canal. **(C)** Additional axial CT image confirming persistent bowel herniation within the right obturator canal prior to interhospital transfer.

However, during transfer to our hospital over a distance of approximately 30 km, the patient's symptoms changed substantially. On arrival, her abdominal pain had resolved completely, and the previously described radiation to the medial thigh was no longer present. Repeat abdominal examination showed a soft, non-distended abdomen without localized tenderness or peritoneal signs. In view of this unexpected clinical improvement, contrast-enhanced abdominal CT was performed to reassess bowel viability and confirm the current status of the previously documented hernia.

The repeat contrast-enhanced CT scan no longer demonstrated a herniated bowel loop within the right obturator canal (Figure 2). The small bowel loops were normally opacified and showed preserved enhancement, without bowel wall thickening, pneumatosis, mesenteric congestion, free fluid, or free intraperitoneal air. No transition point or features of mechanical small bowel obstruction were identified. The appendix was normal in caliber, and no secondary inflammatory changes were noted. Incidental findings included mild dilatation of the left ovarian vein and chronic multilevel vertebral compression deformities, without clinical relevance to the acute presentation. Taken together, the discrepancy between the initial CT findings and the repeat contrast-enhanced CT, along with complete resolution of symptoms, was interpreted as spontaneous reduction of a right obturator hernia.

**Figure 2:**
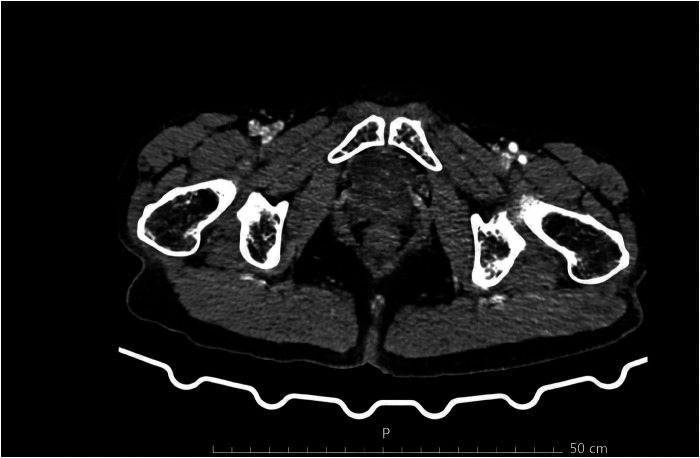
Follow-up contrast-enhanced CT documenting complete spontaneous reduction of the previously incarcerated obturator hernia, illustrating the dynamic radiologic evolution demonstrated in this case.

Although the acute strangulation event had apparently resolved, the initial imaging evidence of a true obturator hernia was considered clinically important. Because the obturator canal is a narrow osteofibrous tunnel and recurrent incarceration may occur unpredictably, conservative observation alone was not considered definitive treatment. The decision to proceed with elective laparoscopic TAPP repair was therefore based on the persistence of the underlying anatomical defect rather than the patient's transient clinical improvement, with the aim of preventing recurrent incarceration and subsequent bowel strangulation. After discussion within the surgical team, the patient was scheduled for elective laparoscopic repair after optimization and close inpatient observation. During the observation period, she remained clinically stable, tolerated oral intake, and had no recurrent abdominal pain, vomiting, fever, or signs of bowel obstruction.

Three days after admission, the patient underwent elective laparoscopic transabdominal preperitoneal repair. Under general anaesthesia, pneumoperitoneum was established and a standard laparoscopic approach was used. Intraoperative exploration revealed no dilated small bowel, no ischemic bowel segment, and no intra-abdominal contamination. Inspection of the right pelvic wall identified a widened right obturator defect, consistent with the site of the previously documented hernia. No bowel loop or omentum was incarcerated within the obturator canal at the time of surgery, confirming that the hernia had reduced before operative intervention (Figure 3). Inspection of the contralateral groin and obturator region revealed no evidence of left-sided obturator hernia or other concomitant groin hernias.

**Figure 3:**
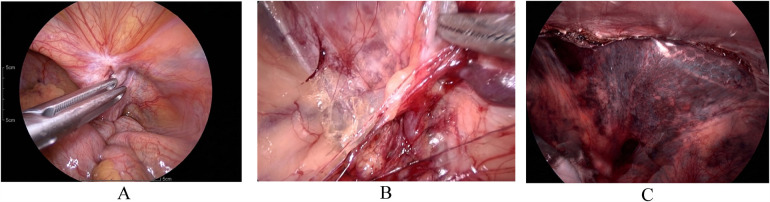
Intraoperative laparoscopic confirmation of the persistent obturator defect and definitive elective TAPP repair following spontaneous hernia reduction. **(A)** Initial laparoscopic exploration demonstrating the right obturator defect after spontaneous reduction, with no residual bowel or omental incarceration. **(B)** Preperitoneal dissection exposing the right obturator canal and surrounding pelvic structures. **(C)** Final laparoscopic view after mesh placement and completion of TAPP repair.

A peritoneal flap was created to expose the preperitoneal space on the right side. Careful dissection was performed to identify the relevant anatomical landmarks and to avoid injury to the obturator vessels and nerve. The myopectineal orifice and adjacent potential hernia sites were inspected. A 10 × 15 cm ULTRAPRO ADVANCED™ (Ethicon, USA) partially absorbable lightweight macroporous mesh was placed to cover the right obturator defect as well as the surrounding weak areas of the groin region. Mesh fixation was performed using a Covidien ProTack™ fixation device, with one tack placed on Cooper's ligament and two additional tacks placed on the anterior abdominal wall to achieve stable fixation. No fixation was applied around the obturator canal because of the close proximity of the obturator vessels and nerve, and fixation within the Triangle of Pain was deliberately avoided to minimize the risk of postoperative neural injury. The peritoneal flap was subsequently closed to isolate the mesh from the abdominal cavity.

The operative time was 45 min. Intraoperative blood loss was minimal, limited to gauze staining only. No bowel resection was required, and no intraoperative complications occurred. The postoperative course was uneventful. On postoperative day 2, the patient was alert and haemodynamically stable, with a soft and non-tender abdomen, dry wounds with only minimal dressing staining, and no palpable groin swelling on either side. The patient recovered well, with no postoperative groin pain, seroma, wound complication, ileus, or recurrent symptoms. She resumed oral intake and mobilization without difficulty. Elective laparoscopic TAPP repair was performed at 9:45 AM on May 6, 2026, and the patient was discharged in stable condition at 3:00 PM on May 8, 2026. The final diagnosis was a spontaneously reduced right obturator hernia treated definitively by elective laparoscopic TAPP mesh repair.

At postoperative day 8, the trocar wounds had healed well and the sutures were removed. At postoperative day 14, the patient remained asymptomatic, with normal bowel and urinary function, no groin pain or swelling, and no clinical or ultrasonographic evidence of recurrent hernia. At this follow-up visit, she expressed satisfaction with the treatment because of minimal postoperative pain, early discharge, and rapid return to her normal daily activities.

## Discussion

Obturator hernia remains one of the most diagnostically challenging entities among abdominal wall hernias because of its rarity, deep pelvic location, and nonspecific clinical presentation. Most patients are elderly, thin, multiparous women with progressive loss of protective preperitoneal fat surrounding the obturator canal, a condition that predisposes the bowel to herniation through this anatomically narrow osteofibrous tunnel. The predominance in frail elderly women has led to the classical description of OH as the “little old lady's hernia”. Delayed diagnosis is common because the hernia is usually not externally visible and the presenting symptoms may resemble orthopedic, neurologic, or nonspecific gastrointestinal disorders. Consequently, many patients present only after the development of bowel obstruction, strangulation, or ischemia, which explains the persistently high morbidity and mortality reported in the literature (1–4).

The present case is clinically notable because the patient exhibited a characteristic but often underrecognized symptom constellation before spontaneous reduction occurred. The initial manifestation of intermittent lower abdominal pain associated with radiation to the medial thigh represented a typical Howship–Romberg sign caused by compression of the obturator nerve within the canal. Although traditionally considered pathognomonic, this sign has been reported inconsistently and may be absent in a substantial proportion of patients, thereby limiting its standalone diagnostic value (5, 6). In our patient, however, the presence of medial thigh pain prompted early imaging evaluation before progression to complete bowel obstruction or peritonitis. This emphasizes that, despite advances in imaging technology, meticulous clinical assessment remains essential in elderly thin women presenting with unexplained abdominal pain and thigh discomfort. Early clinical suspicion may significantly alter outcomes by allowing intervention before bowel compromise develops. A similar diagnostic challenge was described by Major et al., who reported that transient clinical improvement may falsely reassure clinicians despite persistence of the underlying obturator defect, emphasizing that symptom fluctuation does not exclude potentially life-threatening pathology (8).

Computed tomography has fundamentally changed the diagnostic landscape of obturator hernia. Before the widespread availability of CT, most cases were diagnosed intraoperatively after emergent laparotomy for intestinal obstruction (7). Modern multidetector CT enables accurate visualization of bowel herniation between the pectineus and obturator muscles, assessment of bowel viability, and identification of associated complications such as closed-loop obstruction or ischemia (9–11). The present case further illustrates an important but rarely emphasized radiologic phenomenon: spontaneous reduction of the hernia before definitive surgery. The initial non-contrast CT clearly demonstrated bowel protrusion through the right obturator canal, whereas repeat contrast-enhanced CT after transfer showed complete disappearance of the herniated bowel loop and restoration of normal bowel passage. Such radiologic discrepancy can easily lead to diagnostic uncertainty or even dismissal of the initial findings if prior imaging is not carefully reviewed. From a pathophysiological perspective, spontaneous reduction may occur because transient changes in intra-abdominal pressure, patient positioning, bowel peristalsis, or relaxation during transportation permit the incarcerated bowel loop to withdraw from the canal (12). Although uncommon, this phenomenon carries important clinical implications because spontaneous reduction does not eliminate the underlying anatomical defect or the risk of recurrent incarceration. Yokoyama et al. demonstrated that preoperative CT substantially improves diagnostic accuracy and postoperative outcomes in obturator hernia by enabling earlier recognition before irreversible bowel ischemia occurs (3). In the present case, sequential CT imaging was particularly valuable because it documented the dynamic transition from incarcerated obturator hernia to spontaneous reduction during interhospital transfer. To our knowledge, only a limited number of reports have clearly demonstrated such radiologic disappearance of the herniated bowel loop on sequential imaging. Beyond its rarity, this sequential radiologic documentation has considerable educational value because it allows clinicians to directly appreciate the dynamic evolution of obturator hernia from incarceration to spontaneous reduction. The combination of sequential CT findings and subsequent laparoscopic confirmation provides objective radiologic–surgical correlation that may facilitate recognition of similar presentations in future clinical practice. We believe this imaging sequence may help reduce diagnostic uncertainty when repeat CT findings appear deceptively normal after previously documented incarceration.

An important lesson from this case is that spontaneous symptom resolution should not be interpreted as definitive recovery. Similarly, apparently normal repeat CT findings should be interpreted cautiously when previous imaging has already documented an incarcerated obturator hernia, as failure to correlate sequential imaging with the clinical history may result in missed diagnosis and delayed surgical treatment. Unlike reducible inguinal hernias, obturator hernias occur within a rigid osseous tunnel with limited capacity for expansion. Once herniation has occurred, repeated incarceration episodes are highly likely, and subsequent episodes may progress rapidly to strangulation. Several reports have documented delayed diagnosis after transient symptom improvement, resulting in bowel necrosis and emergency bowel resection (13, 14). Therefore, the presence of prior imaging-confirmed obturator hernia should itself be regarded as a strong indication for definitive surgical repair, even if the patient becomes asymptomatic and repeat imaging no longer demonstrates incarceration. In our case, elective surgery after short-term observation allowed definitive treatment under optimized conditions while avoiding the morbidity associated with emergency laparotomy and bowel resection. Matsutani et al. similarly reported that delayed elective laparoscopic repair after successful reduction of incarcerated obturator hernia may represent a safe and effective strategy in clinically stable patients without signs of bowel ischemia (15). Their experience, together with our case, supports the concept that temporary spontaneous reduction should not preclude definitive surgical treatment because the underlying hernia defect persists and remains vulnerable to recurrent strangulation.

The optimal surgical approach for obturator hernia remains debated because most available evidence derives from retrospective case series and isolated reports. Traditionally, lower midline laparotomy has been preferred in emergency settings because it permits rapid exploration of the bowel and facilitates intestinal resection when ischemia is present. However, laparoscopic techniques have increasingly gained acceptance in hemodynamically stable patients and in cases diagnosed before irreversible bowel compromise (16–18). Among minimally invasive techniques, the transabdominal preperitoneal (TAPP) approach offers several important advantages. It provides excellent visualization of the obturator canal and adjacent myopectineal orifice, permits inspection of bowel viability, and allows identification of concomitant groin hernias that may coexist bilaterally. In addition, laparoscopic surgery is associated with reduced postoperative pain, faster mobilization, shorter hospitalization, and lower pulmonary morbidity, particularly beneficial in elderly frail patients (19). In the present case, laparoscopic TAPP repair enabled precise identification of the obturator defect after spontaneous reduction and facilitated broad mesh reinforcement of the entire weakened groin region without the need for bowel manipulation or resection. Recent reports by Chan et al. and Shah et al. further support the expanding role of laparoscopic TAPP repair in obturator hernia, particularly in patients diagnosed before bowel necrosis develops (20, 21). These authors emphasized the superior visualization of the pelvic floor and the ability to evaluate concomitant groin defects while minimizing surgical trauma in elderly patients. Similarly, our patient benefited from definitive minimally invasive repair under optimized elective conditions, thereby avoiding bowel resection and prolonged postoperative recovery. More importantly, spontaneous reduction did not alter our surgical indication because the underlying obturator defect remained intact and continued to pose a substantial risk of recurrent incarceration. Once bowel viability had been confirmed, elective laparoscopic TAPP repair provided definitive anatomical correction while preserving all the advantages of minimally invasive surgery.

Mesh reinforcement during elective repair deserves particular consideration. Primary suture closure alone may be technically feasible in contaminated or emergency settings, but recurrence remains a concern because the surrounding tissues are frequently attenuated in elderly patients (22). In contrast, prosthetic mesh placement during clean elective surgery provides durable reinforcement of the obturator canal and adjacent pelvic floor defects. In the present case, a lightweight partially absorbable mesh was selected to provide broad preperitoneal reinforcement while minimizing foreign-body burden, and fixation was deliberately kept away from the obturator canal to reduce the risk of obturator nerve injury. Our patient underwent successful mesh-based TAPP repair with an uneventful postoperative recovery and no early recurrence-related symptoms. Beyond the individual technical success, this case contributes to the limited literature regarding spontaneously reduced obturator hernia managed electively with minimally invasive mesh repair. It underscores three clinically relevant principles: first, careful correlation with prior imaging is essential even when repeat imaging appears normal; second, spontaneous reduction does not eliminate the need for definitive surgery; and third, laparoscopic TAPP repair represents a rational and effective strategy for stable patients diagnosed before bowel ischemia develops. Most importantly, this case demonstrates a rare radiologic–clinical dissociation in which an initially incarcerated obturator hernia became completely unapparent on repeat imaging despite persistence of the underlying defect. Recognition of this phenomenon is essential to avoid missed diagnosis and delayed definitive treatment. A limitation of the present report is the relatively short postoperative follow-up, which precludes assessment of long-term recurrence and chronic postoperative pain. Nevertheless, the available early clinical and ultrasonographic follow-up demonstrated an uneventful recovery without evidence of recurrence.

## Conclusion

This case highlights a rare presentation of obturator hernia with spontaneous preoperative reduction documented by sequential CT imaging. The discrepancy between the initial CT findings and the subsequent normal contrast-enhanced CT illustrates the diagnostic challenge of spontaneously reduced obturator hernia and emphasizes the importance of correlating sequential imaging with the clinical history, particularly in elderly thin women presenting with abdominal pain and a Howship–Romberg sign. Spontaneous symptom resolution should not be interpreted as definitive recovery because the underlying obturator defect persists and remains at risk for recurrent incarceration and strangulation. The sequential CT findings, together with laparoscopic confirmation, provide valuable educational insight into this uncommon clinical phenomenon. Elective laparoscopic TAPP mesh repair offers definitive anatomical correction while preserving the advantages of minimally invasive surgery and should be considered in clinically stable patients after bowel viability has been confirmed, even when spontaneous reduction has occurred.

## Data Availability

The raw data supporting the conclusions of this article will be made available by the authors, without undue reservation.
